# Individual associations of self-reported sleep duration, sleep quality, chronotype and social jet lag with infectious disease risk

**DOI:** 10.1098/rstb.2023.0472

**Published:** 2025-01-23

**Authors:** Estefanía Martínez-Albert, Josef J. Bless, Luciana Besedovsky

**Affiliations:** ^1^ Institute of Medical Psychology, LMU Munich, Munich 80336, Germany; ^2^ Institute of Medical Psychology and Behavioral Neurobiology, University of Tübingen, Tübingen 72076, Germany

**Keywords:** sleep, chronotype, social jet lag, common cold, infection

## Abstract

Sleep deficiency is associated with infectious disease risk. However, little is known about the individual roles of different aspects of sleep, including sleep duration, sleep quality, sleep timing (assessed by chronotype) and sleep regularity (in the form of social jet lag) in this context. Here, we examined associations of the probability of reporting a cold or other infections with self-reported sleep duration, sleep quality and chronotype in a sample of 642 adults, and with social jet lag in a subsample of 274 adults. We found that short (≤ 6 h) and long sleepers (≥ 9 h) were more likely to report a cold in the past 30 days than average sleepers (7–8 h). Also, individuals with a definite evening chronotype were more likely to report a cold in the past 30 days than those with an intermediate chronotype, even when controlling for sleep duration. Finally, social jet lag was dose-dependently associated with the risk of reporting a cold in the past 12 months, independently of sleep duration, sleep quality and chronotype. No associations were found with sleep quality or with infections other than colds. The findings show that different aspects of sleep are independently associated with incidence of reported colds.

This article is part of the Theo Murphy meeting issue ‘Circadian rhythms in infection and immunity’.

## 1. Introduction

Approximately one in four Europeans experience sleep problems, sleep disturbances, or disorders such as insomnia [1,2]. Sleep disturbances can contribute to a significant health burden, impacting both physical health and mental well-being [3]. The impact of sleep on human health is extensive, influencing various physiological and cognitive functions [4]. Among other factors, sleep plays a crucial role in maintaining a competent immune system, and sleep deficiency has been associated with compromised immune function and reduced (self-reported) immune health status [5–7].

Viral and bacterial infections are considered a major global health threat [8]. In Europe, common viral infections include respiratory viruses, such as those causing the common cold (mainly rhinoviruses [9]), the influenza virus causing the flu [10] and SARS-CoV2 causing COVID-19 [11]. Bacterial infections are also common and can induce infections of the urinary tract, gastrointestinal system, respiratory tract (such as pneumonia and bronchitis), and skin and soft tissue [12]. Short sleep duration has been specifically linked with an increased risk of respiratory infections [13–15]. However, the role of other sleep parameters, including sleep quality, sleep timing and sleep regularity, for the susceptibility to acute infections is less clear. It is also not known whether these sleep parameters are independently associated with infectious disease risk. Sleep quality refers to how well a person sleeps, the depth of their sleep cycles and how restorative their sleep is. It includes factors such as fragmentation of sleep, the efficiency of sleep (i.e. the percentage a person is asleep while lying in bed), and the overall subjective experience to sleep [16]. However, people do not only differ in sleep duration and quality. There are also large individual differences in the timing of sleep as well as how regular this timing is. The chronotype reflects an individual’s natural preference for a particular time of day, determining when the person feels most alert and active. In this context, people are categorized as morning types (‘early birds’), evening types (‘night owls’), or somewhere in between [17]. Research suggests that chronotype affects overall health [18], although the relationship is complex, because chronotype is closely linked to sleep–wake patterns [19]. Also, irregular sleep patterns, such as occurring in shift workers, have been associated with compromised health [20] and recently also with increased incidence of reported colds [21]. Another example of irregular sleep timing is social jet lag, which describes the misalignment between an individual’s natural circadian rhythm and their socially imposed sleep schedule, leading to different sleep times during workdays and work-free days [22]. The incidence of social jet lag in the general population is very high and, even if the shift in sleep timing is usually less pronounced than in shift workers, it has been associated with a range of health issues, including depression, obesity and cardiovascular problems [23–25]. Nonetheless, the role of chronotype and social jet lag in infectious disease risk is not clear so far.

The aim of this study was to examine the individual associations between different self-reported sleep parameters (i.e. sleep duration, sleep quality, sleep timing as assessed with chronotype and social jet lag reflecting poor sleep regularity) and the probability of reported colds and other infections in adults.

## 2. Methods

### (a) Participants and study design

Participants were 642 adults from Germany and Spain who were recruited via advertisements and completed an online survey or paper-based questionnaires from August 2017 to July 2023. All participants were 18 years or older at the moment of enrolment in the study. The need for institutional review board approval was waived by the Universities of Tübingen and Munich, Germany, because the data was collected anonymously.

### (b) Survey items

The survey consisted of several questionnaires asking about demographic variables, previous infections, sleep parameters, as well as perceived health and stress as described in detail in the following. The country where and the season when the survey was completed (Germany or Spain; winter, spring, summer, autumn) were also recorded.

#### (i) Participants’ characteristics

Participants reported demographic variables, including age (mean ± s.d. 27.29 ± 10.15 years) and sex (female; male; no answer) and also reported on their health and stress perception during the past 30 days and 12 months by filling out two rating scales (from 1 to 5) with the following questions: ‘How would you rate your average health over the past 30 days / past 12 months?’, and ‘How stressed have you felt in the past 30 days / past 12 months?)’, where 1 corresponded to ‘very good health’ or ‘not stressed at all’, and 5 corresponded to ‘very bad health’ or ‘very stressed’. Participants also reported whether a physician has ever diagnosed them with a sleep disorder, and if yes, which sleep disorder, and if they had ever told a physician that they had trouble sleeping.

#### (ii) Colds and infections

Participants reported whether they had experienced a cold (head or chest) or another infection, including influenza (flu), pneumonia, ear infection and COVID-19 (for the subgroup surveyed during the pandemic) in the past 30 days and 12 months.

#### (iii) Sleep duration

Typical weekday sleep duration was assessed with the question: ‘What is your typical sleep duration during the week (in hours)?’ like in the study by Prather & Leung [13]. In case the participants also indicated decimal numbers, the responses were rounded to the nearest hour. For half hours (e.g. 6.5 h), they were rounded to the nearest previous hour (e.g. 6 h). Sleep duration was then stratified as short sleep (≤ 6 h), average sleep (7–8 h) and long sleep (≥ 9 h). Since in the literature different ways of categorization were used, we performed exploratory analyses with different categories (e.g. short sleep determined as < 6 h) and other ways of rounding (e.g. rounding half hours to the nearest following hour). This did not significantly change the results (data not shown).

#### (iv) Sleep quality

Sleep quality was assessed with the Pittsburgh Sleep Quality Index (PSQI) [26], a 19-item self-rated questionnaire, which assesses different components of subjective sleep quality retrospectively (for the past four weeks). The items are grouped into seven components (i.e. subjective sleep quality, sleep latency, sleep duration, sleep efficiency, sleep disturbances, use of sleep medications and daytime dysfunction), which are all strongly inter-correlated and are thought to reflect different aspects of the same overall construct [26]. The overall score ranges from 0 to 21 points, with scores ≤ 5 points indicating good sleep quality and scores > 5 points indicating poor sleep quality.

#### (v) Chronotype

Chronotype was determined with the Morningness-Eveningness questionnaire (MEQ) [27]. This scale assesses individual differences in morningness and eveningness in human circadian rhythms with questions about daily activities and sleep–wake patterns. Among other questions, participants are asked to indicate at what time they would get up/go to bed if they were entirely free to plan their day/evening, how easy they find it to get up in the morning, how alert/hungry/tired they feel during the first half-hour after they wake up in the morning, at what time of the day they feel their best in terms of alertness and energy levels, at what time they would prefer to take an important exam or test. To obtain a global score, the 19 items of the scale are summed up and categorized into 5 groups: definite morning type (70–86 points), moderate morning type (59–69), neither type (42–58, referred to as ‘intermediate type’ in the following), moderate evening type (31–41) and definite evening type (16–30).

#### (vi) Social jet lag

Social jet lag was evaluated using the Munich Chronotype Questionnaire (MCTQ) [28]. The MCTQ is a self-rated scale that determines the individual phase of entrainment on work and work-free days. Social jet lag is assessed as the difference in the midpoint between sleep on- and offset on workdays and free days (in minutes). The following categories were built: no social jet lag (0–15 min), moderate social jet lag (16–60 min) and pronounced social jet lag (> 60 min).

### (c) Statistical analysis

We collected data from 664 participants. Of these, 22 participants were excluded from the analysis because of missing data in at least one of the main outcomes (i.e. colds or infections during the past 30 days or past 12 months), yielding a final sample size of 642 participants. Social jet lag was assessed in a subgroup of 274 participants.

Data analyses were conducted with SPSS, version 28 (IBM SPSS Statistics). The associations between sleep variables (i.e. sleep duration, sleep quality, chronotype and social jet lag) and colds or other infections during the past 30 days or past 12 months were explored using multivariable logistic regression models. For analyses of sleep duration, individuals reporting ‘7–8 hours’ of sleep were considered the reference group. For analyses of sleep quality, individuals presenting ‘good sleep quality’ (i.e. a global PSQI score ≤ 5) were treated as the reference group. For chronotype, individuals categorized as ‘intermediate’ on the MEQ scale were treated as the reference group. Finally, for analyses of social jet lag, individuals presenting ‘no social jet lag’ (i.e. 0–15 min) were considered the reference group.

An initial baseline model was constructed entering the sleep variable of interest while adjusting for age, sex, country of the survey and season. Because the different sleep parameters may influence each other (e.g. participants with an evening chronotype often have a shorter sleep duration compared with intermediate types) [19], in further models, we additionally adjusted for the respective other sleep parameters (except for social jet lag, because we only had data from a subgroup of participants for this measure). After adjustment, the risk of each outcome was calculated and expressed as odds ratios (OR) with 95% confidence intervals (CIs). Two-sided *p*-values < 0.05 were considered statistically significant. Since data collection was carried out in a period before and during the COVID-19 pandemic, we performed additional analyses, in which we also controlled for ‘pandemic status’ (i.e. participation in the study before the World Health Organization declared COVID-19 as a pandemic on 11 March 2020, or after this date). Adding this variable to the models did not significantly change the results (data not shown).

## 3. Results

A total of 642 participants were included in the study. Their characteristics are presented in table 1. A subsample of 274 participants additionally filled in the MCTQ to assess social jet lag. The characteristics of this subsample are shown in the electronic supplementary material, table S1.

**Table 1.** Descriptive statistics of total study sample.

	number		percentage (%)	
**sex**				
female	356		55.45	
male	284		44.24	
no answer	2		0.31	
**age (years)**				
18–25	369		57.48	
26–40	207		32.24	
> 40	66		10.28	
**country of survey**				
Germany	521		81.15	
Spain	121		18.85	
**sleep disorder**				
no	628		97.82	
yes	14		2.18	
**sleep duration**				
short sleepers (≤ 6 h)	164		25.55	
average sleepers (7–8 h)	453		70.56	
long sleepers (≥ 9 h)	25		3.89	
**sleep quality**				
good quality	515		80.22	
poor quality	127		19.78	
**chronotype**				
definite morning type	12		1.87	
moderate morning type	96		14.95	
intermediate type	342		53.27	
moderate evening type	144		22.43	
definite evening type	48		7.48	
**social jet lag** ^a^				
0–15 min	20		7.30	
16–60 min	130		47.45	
> 60 min	124		45.26	

self-reported infections, health perception and stress				
	past 30 days		past 12 months	
	number	percentage (%)	number	percentage (%)
**common cold**				
yes	195	30.37	571	88.94
no	447	69.63	71	11.06
**other infections**				
yes	37	5.76	186	28.97
no	605	94.24	456	71.03
**health perception**				
1 (very good)	214	33.33	172	26.79
2	273	42.52	295	45.95
3	101	15.73	110	17.13
4	45	7.01	56	8.72
5 (very bad)	9	1.40	9	1.40
**stress perception**				
1 (not stressed at all)	42	6.54	32	4.98
2	184	28.66	138	21.50
3	176	27.41	233	36.29
4	176	27.41	184	28.66
5 (very stressed)	64	9.97	55	8.57

^a^ Social jetlag analyses were performed in a subsample of 274 participants.

### (a) Relationship between sleep variables and reports of colds

**Figure 1.**
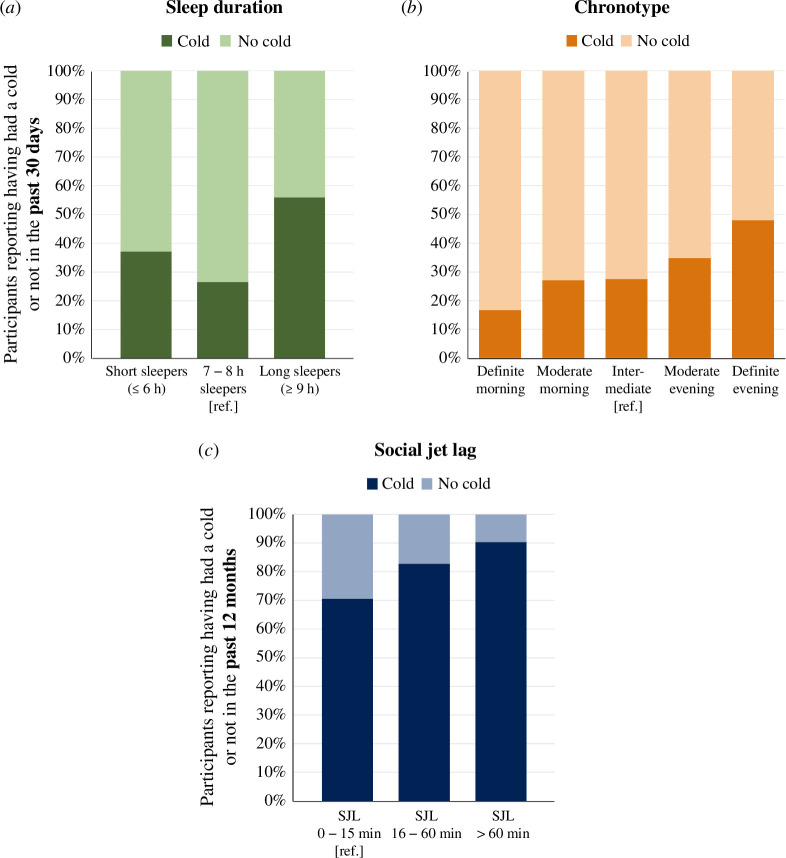
Sleep duration, chronotype and social jet lag are associated with the likelihood of reporting a cold. (*a*) Percentage of participants who reported having had a cold (dark green) or not (light green) in the past 30 days, stratified by sleep duration (≤ 6 h, between 7 and 8 h, and ≥ 9 h); (*b*) percentage of individuals who reported having had a cold (dark orange) or not (light orange) in the past 30 days, stratified by chronotype (definite morning type, moderate morning type, intermediate type, moderate evening type and definite evening type); (*c*) percentage of participants who reported having had a cold (dark blue) or not (light blue) in the past 12 months, stratified by the amount of social jet lag (SJL; 0–15 min, 16–60 min and > 60 min).

Compared with participants who reported sleeping 7–8 h per night, both short sleepers (≤ 6 h of sleep per night) and long sleepers (≥ 9 h of sleep per night) were significantly more likely to report a cold in the past 30 days, independently of age, sex, survey season and country (OR = 1.83, 95% CI 1.21–2.76 for short sleepers; OR = 3.6, 95% CI 1.52–8.57 for long sleepers) (figure 1). These associations were independent of sleep quality (OR = 1.86, 95% CI 1.20–2.88 for short sleepers; OR = 3.61, 95% CI 1.52–8.59 for long sleepers) and chronotype (OR = 1.75, 95% CI 1.16–2.65 for short sleepers; OR = 3.24, 95% CI 1.36–7.72 for long sleepers) (table 2). Subjective sleep duration was not significantly associated with the likelihood of reporting a cold in the past 12 months.

**Table 2.** Associations of sleep duration, sleep quality, chronotype and social jet lag with self-reported colds in the past 30 days and 12 months.

		model 1^b^		model 2^c^		model 3^d^		model 4^e^	
	colds in past 30 days, *N* (%)	OR (95% CI)	*p*‐value	OR (95% CI)	*p*‐value	OR (95% CI)	*p*‐value	OR (95% CI)	*p*‐value
**sleep duration:**									
short sleepers (≤ 6 h)	61 (37.2)	1.83(1.21–2.76)	**0**.**004**			1.86(1.20–2.88)	**0**.**006**	1.75(1.16–2.65)	**0.008**
average sleepers (7–8 h)	120 (26.5)	1 [Ref.]				1 [Ref.]		1 [Ref.]	
long sleepers (≥ 9 h)	14 (56)	3.6(1.52–8.57)	**0.004**			3.61(1.52–8.59)	**0.004**	3.24(1.36–7.72)	**0.008**
**sleep quality:**									
good quality	148 (28.7)	1 [Ref.]		1 [Ref.]				1 [Ref.]	
poor quality	47 (37)	1.14(0.80–1.62)	0.484	1.05(0.72–1.52)	0.816			1.05(0.73–1.51)	0.797
**chronotype:**									
definite morning	2 (16.7)	0.44(0.09–2.14)	0.311	0.47(0.10-2.27)	0.348	0.45(0.09-2.15)	0.315		
moderate morning	26 (27.1)	1.03(0.61–1.74)	0.915	1.03(0.61–1.75)	0.907	1.03(0.61–1.75)	0.902		
intermediate type	94 (27.5)	1 [Ref.]		1 [Ref.]		1 [Ref.]			
moderate evening	50 (34.7)	1.24(0.79–1.94)	0.344	1.24(0.80–1.94)	0.339	1.23 (0.79-1.93)	0.362		
definite evening	23 (47.9)	2.12(1.11–4.04)	**0.023**	2.07(1.08–3.96)	**0.029**	2.09 (1.09-4.02)	**0.027**		
**social jet lag** ^a^ **:**									
0–15 min	10 (29.4)	1 [Ref.]		1 [Ref.]		1 [Ref.]		1 [Ref.]	
16–60 min	26 (22.4)	0.75(0.30–1.85)	0.532	0.74(0.30–1.84)	0.516	0.73(0.29–1.81)	0.498	0.70(0.28–1.74)	0.438
> 60 min	40 (32.3)	0.99(0.41–2.37)	0.984	0.98(0.41–2.36)	0.960	0.99(0.42–2.39)	0.991	0.87(0.35–2.17)	0.770

	colds in past 12 months, N (%)	OR (95% CI)	*p*‐value	OR (95% CI)	*p*‐value	OR (95% CI)	*p*‐value	OR (95% CI)	*p*‐value
**sleep duration:**									
short sleepers (≤ 6 h)	152 (92.7)	1.51(0.79–2.85)	0.210			1.40(0.71–2.74	0.334	1.4(0.74–2.69)	0.299
average sleepers (7–8 h)	396 (87.4)	1 [Ref.]				1 [Ref.]		1 [Ref.]	
long sleepers (≥ 9 h)	23 (92)	1.65(0.36–7.47)	0.513			1.62(0.36–7.32)	0.530	1.28(0.28–5.84)	0.749
**sleep quality:**									
good quality	452 (87.8)	1 [Ref.]		1 [Ref.]				1 [Ref.]	
poor quality	119 (93.7)	1.50(0.84–2.66)	0.168	1.32(0.73–2.39)	0.363			1.35(0.76–2.43)	0.312
**chronotype:**									
definite morning	9 (75)	0.27(0.06–1.12)	0.072	0.30(0.07–1.24)	0.096	0.29(0.07–1.19)	0.086		
moderate morning	82 (85.4)	0.61(0.32–1.18)	0.144	0.63(0.33–1.22)	0.169	0.63(0.33–1.22)	0.169		
intermediate type	305 (89.2)	1 [Ref.]		1 [Ref.]		1 [Ref.]			
moderate evening	130 (90.3)	0.87(0.45–1.69)	0.685	0.88(0.45–1.70)	0.697	0.86(0.44–1.66)	0.646		
definite evening	45 (93.8)	1.64(0.47–5.79)	0.435	1.69(0.48–5.98)	0.417	1.58(0.45–5.60)	0.475		
**social jet lag** ^a^ **:**									
0–15 min	24 (70.6)	1 [Ref.]		1 [Ref.]		1 [Ref.]		1 [Ref.]	
16–60 min	96 (82.8)	3.11(1.14–8.50)	**0.027**	3.08(1.13–8.42)	**0.028**	3.12(1.14–8.53)	**0.026**	2.80(1.00–7.85)	**0.050**
> 60 min	112 (90.3)	4.28(1.50–12.21)	**0.007**	4.22(1.48–12.06)	**0.007**	4.30(1.50–12.27)	**0.007**	3.60(1.19–10.84)	**0.023**

^a^ Social jetlag analyses were performed in a subsample of 274 participants. ‘Country’ was not added for social jetlag because all participants were from Germany.

^b^ Model adjusted for sex, age, country, season

^c^ Model adjusted for sex, age, country, season, sleep duration

^d^ Model adjusted for sex, age, country, season, sleep quality

^e^ Model adjusted for sex, age, country, season, chronotype

Sleep quality, as assessed using the global PSQI score, was neither associated with the likelihood of reporting a cold during the past 30 days nor the past 12 months in any of the models studied (table 2).

Individuals falling into the category of a ‘definite evening chronotype’ were more likely to report a cold during the past 30 days than those who presented an ‘intermediate chronotype’ (OR = 2.12, 95% CI 1.11–4.04) (figure 1). This association remained significant after adjusting for sleep duration (OR = 2.07, 95% CI 1.08–3.96) or sleep quality (OR = 2.09, 95% CI 1.09–4.02) (table 2). Chronotype was not significantly associated with the likelihood of reporting a cold during the past 12 months.

Social jet lag was not significantly associated with likelihood of reporting a cold during the past 30 days (table 2). However, participants with a social jet lag of 16–60 min were more likely to report a cold in the past 12 months compared with participants without social jet lag (OR = 3.11, 95% CI 1.14–8.50). This association was even more pronounced for participants with > 60 min of social jet lag (OR = 4.28, 95% CI 1.50–12.21) (figure 1). These effects were still evident after adjusting for sleep duration (OR = 3.08, 95% CI 1.13–8.42 and OR = 4.22, 95% CI 1.48–12.06, respectively, for those with 16–60 min and > 60 min of social jetlag), sleep quality (OR = 3.12, 95% CI 1.14–8.53 and OR = 4.30, 95% CI 1.50–12.27, respectively, for those with 16–60 min and > 60 min of social jet lag) or chronotype (OR = 2.80, 95% CI 1.00–7.85 and OR = 3.60, 95% CI 1.19–10.84, respectively, for those with 16–60 min and > 60 min of social jet lag) (table 2).

### (b) Relationship between sleep variables and reports of other infections

None of the sleep variables studied were associated with the likelihood of reporting an infection other than a cold, neither in the past 30 days nor the past 12 months in any of the models studied (table 3).

**Table 3.** Associations of sleep duration, sleep quality, chronotype and social jetlag with other self-reported infections during the past 30 days and 12 months.

		model 1^b^		model 2^c^		model 3^d^		model 4^e^	
	infections in past 30 days, *N* (%)	OR (95% CI)	***p*** **‐value**	OR (95% CI)	***p*** **‐value**	OR (95% CI)	***p*** **‐value**	OR (95% CI)	***p*** **‐value**
**sleep duration:**									
short sleepers (≤ 6 h)	13 (7.9)	1.31(0.62–2.75)	0.482			1.50(0.66–3.39)	0.331	1.33(0.63–2.81)	0.457
average sleepers (7–8 h)	23 (5.1)	1 [Ref.]				1 [Ref.]		1 [Ref.]	
long sleepers (≥ 9 h)	1 (4)	0.73(0.09–5.77)	0.764			0.72(0.09–5.70)	0.754	0.76(0.09–6.03)	0.792
**sleep quality:**									
good quality	24 (4.7)	1 [Ref.]		1 [Ref.]				1 [Ref.]	
poor quality	13 (10.2)	1.18(0.60–2.33)	0.621	1.17(0.56–2.42)	0.681			1.21(0.61–2.40)	0.582
**chronotype:**									
definite morning	0 (0)	0.00 (0.00-)	0.999	0.00 (0.00-)	0.999	0.00 (0.00-)	0.999		
moderate morning	4 (4.2)	0.66(0.22–1.99)	0.460	0.66(0.22–1.98)	0.456	0.65(0.22–1.98)	0.452		
intermediate type	23 (6.7)	1 [Ref.]		1 [Ref.]		1 [Ref.]			
moderate evening	7 (4.9)	0.64(0.26–1.60)	0.341	0.64(0.26–1.61)	0.344	0.65(0.26–1.64)	0.362		
definite evening	3 (6.3)	0.79(0.22–2.86)	0.723	0.77(0.21–2.83)	0.702	0.82(0.23–3.02)	0.771		
**social jet lag** ^a^ **:**									
0–15 min	4 (11.8)	1 [Ref.]		1 [Ref.]		1 [Ref.]		1 [Ref.]	
16–60 min	5 (4.3)	0.36(0.09–1.47)	0.154	0.39(0.09–1.64)	0.199	0.36(0.09–1.49)	0.160	0.46(0.11–1.96)	0.294
> 60 min	6 (4.8)	0.32(0.08–1.26)	0.103	0.36(0.09–1.42)	0.144	0.32(0.08–1.25)	0.102	0.47(0.11–1.96)	0.298

	infections in past 12 months, N (%)	OR (95% CI)	*p*‐value	OR (95% CI)	*p*‐value	OR (95% CI)	*p*‐value	OR (95% CI)	*p*‐value
**sleep duration:**									
short sleepers (≤ 6 h)	55 (33.5)	1.23(0.81–1.85)	0.328			1.08(0.70–1.69)	0.721	1.17(0.78–1.77)	0.449
average sleepers (7–8 h)	125 (27.6)	1 [Ref.]				1 [Ref.]		1 [Ref.]	
long sleepers (≥ 9 h)	6 (24)	0.75(0.29–1.96)	0.558			0.75(0.30–1.96)	0.558	0.67(0.25–1.75)	0.410
**sleep quality:**									
good quality	134 (26)	1 [Ref.]		1 [Ref.]				1 [Ref.]	
poor quality	52 (40.9)	1.25(0.87–1.79)	0.222	1.26(0.87–1.84)	0.223			1.18(0.82–1.70)	0.377
**chronotype:**									
definite morning	1 (8.3)	0.24(0.03–1.97)	0.186	0.25(0.03–1.98)	0.187	0.25(0.03–2.03)	0.195		
moderate morning	21 (21.9)	0.74(0.43–1.29)	0.287	0.74(0.43–1.29)	0.288	0.76(0.44–1.32)	0.324		
intermediate type	97 (28.4)	1 [Ref.]		1 [Ref.]		1 [Ref.]			
moderate evening	50 (34.7)	1.30(0.82–2.02)	0.250	1.30(0.83–2.02)	0.250	1.25(0.80–1.96)	0.321		
definite evening	17 (35.4)	1.28(0.66–2.49)	0.462	1.28(0.66–2.49)	0.467	1.19(0.61–2.34)	0.611		
**social jet lag** ^a^ **:**									
0–15 min	9 (26.5)	1 [Ref.]		1 [Ref.]		1 [Ref.]		1 [Ref.]	
16–60 min	10 (8.6)	1.14(0.47–2.76)	0.766	1.12(0.46–2.73)	0.794	1.12(0.46–2.71)	0.803	1.09(0.45–2.68)	0.847
> 60 min	10 (8.1)	1.00(0.42–2.41)	0.997	0.98(0.41–2.38)	0.969	1.00(0.42–2.40)	0.999	0.93(0.37–2.31)	0.869

^a^ Social jet lag analyses were performed in a subsample of 274 participants. ‘Country’ was not added for social jet lag because all participants were from Germany.

^b^ Model adjusted for sex, age, country, season

^c^ Model adjusted for sex, age, country, season, sleep duration

^d^ Model adjusted for sex, age, country, season, sleep quality

^e^ Model adjusted for sex, age, country, season, chronotype

## 4. Discussion

We show here that self-reported short or long sleep duration, a ‘definite evening’ chronotype, or experiencing social jet lag are associated with a greater risk of reporting a cold. Importantly, these different sleep parameters were all independently associated with the likelihood of suffering from a cold, suggesting that different mechanisms may underlie these associations.

Regarding self-reported short sleep duration, our findings are consistent with prior studies showing that people with a habitual sleep duration of 5 h or less were more likely to report a cold in the past 2 weeks or 30 days or a general predisposition for common colds, compared with participants sleeping 7 or 7–8 h [13,21,29]. Of note, similar results were found in experimental studies where participants were challenged with a rhinovirus and monitored for the development of a clinical cold [30,31]. Sleep duration was measured either by self-report or wrist actigraphy in these laboratory-based studies. They showed that people with a short sleep duration were more likely to develop a clinically verified cold compared with the reference group sleeping more than 7 h, indicating that our findings, which were based on self-reports of colds, are also seen with objective measures of cold symptoms. A recent meta-analysis including studies with both self-reported as well as objectively assessed sleep duration confirmed the association between short sleep duration and upper respiratory infections [32]. Short habitual sleep duration and experimental sleep deprivation are linked with changes in the number and function of various immune parameters [33–35]. Of note, sleep loss induces an increase in inflammatory cytokines and other mediators of symptoms typically released in response to a viral infection [5,36]. These direct effects of lack of sleep on the immune system may explain the increased risk of reporting a cold in participants with short sleep duration.

Our finding that also self-reported long sleep duration is associated with an increased risk of reporting a cold in the past 30 days is likewise in line with previous studies investigating reports of colds or infections in general in the past 2 weeks or 3 months, respectively [21,37]. However, it is important to mention that it is unclear whether this association reflects a causal effect of reported long sleep duration on infectious disease risk or whether it might be driven by confounding factors that impact sleep behaviours. Specifically, long sleep duration could be a sign of an underlying and potentially previously unrecognized (sub)clinical disease, such as depression [38] or sleep apnea [39]. Long habitual sleep duration is associated with higher systemic inflammatory markers, like C-reactive protein and interleukin-6 [40–42], and this low-grade inflammation may increase susceptibility to infections as well as sleep duration [29,43,44]. In addition, self-reported sleep duration may be confounded with time spent in bed rather than reflecting actual time asleep [45,46], which could also bias the associations. Therefore, further studies employing more precise and objective methods to quantify sleep duration and controlling for subclinical or previously unrecognized disorders are essential to better understand the associations between long sleep duration and infectious disease risk.

In the present study, sleep quality as assessed with the PSQI was not associated with reports of colds or infections. There is some discrepancy in the literature regarding the association between sleep quality and risk of colds/respiratory infections, which may be partly explained by the various ways of assessing sleep quality [32]. Some studies examined the associations between self-reported sleep efficiency and risk of developing a cold. Two of these studies found that individuals with lower self-reported sleep efficiency had a higher risk of developing a clinical cold after experimental rhinovirus exposure [30,47], while another study found no such association [31]. Also, the percentage of nights that participants reported feeling rested after awakening in the morning was not associated with susceptibility to a clinical cold after viral challenge [30]. Another study measured sleep quality using two categories (quite well/well, or neither well nor bad/quite bad/bad) and similarly found no association with reports of upper respiratory infections assessed prospectively during a 9 month follow-up period [48]. In the present study, we focused on the global PSQI score for the assessment of sleep quality, because it is a composite score combining several aspects of the overall construct reflecting sleep quality [26] and can therefore be assumed to be a more robust measure than a single question, and because it is recommended for population-level assessment of sleep quality [49]. However, also with this measure, we did not find an association between subjective sleep quality and reported colds. A recent meta-analysis investigating associations between sleep quality and COVID-19 found an increased risk of COVID-19 infection in those with poor sleep quality [50]. However, the increase was quite small (OR: 1.16), potentially explaining the discrepancy in the literature mentioned above. Sleep quality might be more related to the severity of infectious disease rather than infection risk, as reported in the meta-analysis. Overall, any association between infectious disease risk and sleep quality seems to be less robust than that with sleep duration and, hence, the role of sleep quality in this context needs further investigation.

A major finding of our study is that people falling under the category of a ‘definite evening’ chronotype had a greater risk of reporting a cold in the past 30 days compared with people with an intermediate chronotype. Evening chronotype has been associated with an increased risk for several diseases and negative conditions, including cardiovascular disease [51], diabetes [52], depression, anxiety, substance abuse [52] and increased symptom burden in patients with immune-mediated inflammatory diseases [53]. To our knowledge, we provide the first evidence of a link between eveningness and susceptibility to the common cold. Of note, the association between chronotype and the risk of reporting a cold remained significant after adjusting for factors that are strongly linked with chronotype, like age and sex [54], seasonal change [55] and latitude [56]. Evening chronotype is also often linked with short sleep duration and poor sleep quality [57]. However, neither sleep duration nor sleep quality mediated the association of eveningness with higher risk of reporting a cold in our study, suggesting that chronotype is an independent risk factor. Previous studies have found increases in inflammatory markers associated with evening chronotypes in young and middle-aged adults [58–60], which provides first evidence of immunological dysregulations in these individuals.

We also found that social jet lag was significantly associated with the likelihood of reporting a cold in the past 12 months and the risk was higher the larger the extent of social jet lag was, suggesting a dose-response relationship. These results are in line with other studies that found associations of shift work or experiencing social jet lag greater than 2 h with an increased risk of testing positive for SARS-CoV-2 [61–63]. Our study adds to these findings by showing that even smaller amounts of social jet lag (i.e. 15–60 min) are associated with an increased risk of reporting a respiratory infection. Social jet lag can be linked with poor and shortened sleep [22]. However, the association we found was independent of sleep duration, sleep quality and chronotype, suggesting that an irregular sleep timing leading to circadian misalignment may be an independent risk factor for infectious disease. Given that the circadian clock regulates a wide range of physiological processes, it is no surprise that circadian misalignment in the form of social jet lag has been linked with various (potentially) negative health conditions, such as increased resting heart rate and cortisol levels [64,65], obesity [24], depression [25] and an adverse metabolic profile [66]. Experimentally induced circadian misalignment has been shown to increase inflammation [67,68]. Furthermore, irregular sleep patterns, as assessed by the standard deviation in sleep duration and sleep onset measured over several days, were associated with increases in the number of leukocytes and some leukocyte subsets, which are considered markers of inflammation [69]. However, literature investigating immunological changes in individuals suffering from social jet lag is scarce. Two previous studies found associations between social jet lag and increases in inflammatory markers in clinical samples [70,71]. Another study found changes in immune parameters related to social jet lag in night-shift workers [72]. However, this study did not find such correlations with social jet lag in daytime workers. Therefore, further research is necessary to investigate the immunological mechanisms underlying the association between social jet lag and infectious disease risk.

Social jet lag has a very high prevalence, affecting two-thirds of the working and studying population in industrialized countries [23]. Work and school schedules play a pivotal role in inducing social jet lag [73]. Of note, the implementation of lockdown measures during the global COVID-19 pandemic forced a large portion of the population to adopt remote work setups, allowing for greater flexibility in working hours. During this time, it was observed that on weekdays, sleep duration increased, and both the onset and offset of sleep were delayed. These changes rendered sleep duration on workdays more comparable with that on free days, thus contributing to a reduction in social jet lag [74–77]. Given our finding that social jet lag is associated with common colds, allowing more flexibility in working hours may reduce periods of sickness absence, which would be advantageous for both employees and employers, as well as for the society in general, e.g. with respect to overwhelmed health care systems.

We did not find associations between any of the sleep parameters measured and the risk of experiencing infections other than head or chest colds. These results are in contrast with other studies that have found associations between self-reported short sleep duration and several types of infections within the past 30 days or 3 months, respectively [6,13]. This discrepancy could be due to the fact that only relatively few participants (6%) reported having had infections other than colds in the past 30 days in our study and therefore these findings have to be interpreted with caution. Of note, our study population clearly differed from these previous studies: the study by Bjorvatn *et al*. [6] recruited participants via a network of general practitioners and the study by Prather & Leung [13] used a nationally representative sample of adults from the US, while our participants were to a large extent university students. As a consequence, the health status and average age of our participants were different from those of the previous studies, which could explain the different findings.

It is worth mentioning that sleep duration and chronotype were found to be associated with reported colds in the past 30 days but not in the past 12 months. The reason for this is likely a ceiling effect, because the participants of the reference groups already had a very high incidence of reported colds in the past 12 months (i.e. > 87 %). Descriptively, short and long sleepers indeed had a slightly higher incidence of reported colds in the past 12 months than average sleepers (92.7 and 92 vs. 87.4 %) and the morningness-eveningness variable appeared to be dose-dependently associated with incidence of reported colds, i.e. the higher the eveningness, the higher the cold incidence, indicating that also for the 12 months period, there seems to be an association between sleep duration or chronotype and reported colds. The mentioned ceiling effect does not apply to the same extent to the social jet lag variable, where only about 70% of the reference group reported a cold during this period, which could explain why we did find an association with reported colds in the past 12 months for this variable. On the other hand, the lack of an association of social jet lag with the 30 days period of experiencing a cold might be due to a lack of statistical power, with probably too few participants (in absolute numbers) reporting a cold in the past 30 days in this subsample.

While our study provides valuable insights into the individual associations between several sleep variables and infectious disease risk, it has some limitations. Firstly, the correlational design does not allow us to establish causal relationships between variables. Hence, based on these findings, we cannot tell whether the assessed sleep parameters influenced the risk of experiencing colds or the other way around, or whether even a third factor might have independently affected both variables. An acute infection will likely affect sleep during the period of sickness. However, given that we asked for the ‘typical’ sleep duration and that a (usually mild) common cold would not be expected to have a long-lasting effect on sleep, we think it is rather unlikely that the associations can be explained by an acute effect of the infection on sleep duration. Chronotype can also be considered a relatively stable variable within a 1 year period and is therefore not very likely affected by an acute infection. Despite the efforts to control for confounding variables, there are still factors not considered in the study that could have influenced the observed associations, such as sleep debt or dietary habits and other lifestyle factors. Our study was performed between 2017 and 2023, thus encompassing both a period before and during the COVID-19 pandemic. The unique circumstances introduced by the pandemic, including changes in lifestyle, stress levels and sleep patterns for a substantial portion of the population, could have influenced the observed associations. Our study was not designed or powered to assess the impact of pandemic-related factors on sleep variables and infectious disease risk. However, adjusting for the period of data collection (i.e. before or during the pandemic) did not change the results, indicating that despite the large impact of the pandemic on the population, the associations between several sleep variables and reported colds remained robust. It is also important to note that all data were based on self-reports and we cannot exclude potential report biases. It is conceivable that such report biases are systematically associated with the assessed sleep variables. For example, given that sleep is important for memory consolidation [78], short sleep duration could be associated with worse memory for the reported infections. However, this would bias the results in the opposite direction (i.e. short sleep would be associated with fewer reported colds) and therefore seems rather unlikely as an explanation for our findings. Finally, given that our sample was relatively small in size and was not representative of the general population, generalization of our findings to other contexts is limited. In particular, almost 90% of our study population were less than 40 years old. Further research with a larger and more representative sample, including individuals of different age groups and health status, is needed to validate and extend the current findings. However, our study demonstrates that associations between various sleep variables and colds are visible even in relatively young adults reporting overall good health and it is likely that such associations are even more pronounced in individuals with compromised immune functions.

In conclusion, our study reveals that different parameters related to sleep, including self-reported sleep duration, chronotype and social jet lag, are associated with the risk of reporting a common cold. Importantly, these factors appear to be independently associated with susceptibility to experiencing colds and therefore different mechanisms likely underlie the individual associations. Future research should focus on identifying the distinct mechanisms driving these relationships.

## Data Availability

The data relevant to the conclusions of this paper have been deposited in Open Science Framework [79]. Supplementary material is available online [80].
